# Effects of light irradiation on essential oil biosynthesis in the medicinal plant *Asarum heterotropoides* Fr. Schmidt var. *mandshuricum* (Maxim) Kitag

**DOI:** 10.1371/journal.pone.0237952

**Published:** 2020-09-24

**Authors:** Zhiqing Wang, Shengyuan Xiao, Yufang Wang, Jiyong Liu, Haiqin Ma, Yingping Wang, Yixin Tian, Wei Hou

**Affiliations:** 1 Laboratory of Cultivation and Breeding of Medicinal Plants, National Administration of Traditional Chinese Medicine, College of Chinese Medicinal Materials, Jilin Agricultural University, Changchun, Jilin, China; 2 State & Local Joint Engineering Research Center of Ginseng Breeding and Application, College of Chinese Medicinal Materials, Jilin Agricultural University, Changchun, Jilin, China; 3 Institute of Special Wild Economic Animals and Plants, Chinese Academy of Agriculture Sciences, Changchun, Jilin, China; Lovely Professional University, INDIA

## Abstract

*Asarum heterotropoides* Fr. var. *mandshuricum* (Maxim) Kitag (Chinese wild ginger) is an important medicinal herb. Essential oil extracted from its roots is the key ingredient and is mainly composed of phenylpropanoid compounds. As a skiophyte plant, light is a crucial factor for *A*. *heterotropoides* var. *mandshuricum* growth and metabolism. To investigate the effects of light irradiation on the essential oil biosynthesis in *A*. *heterotropoides* var. *mandshuricum*, the plants were cultivated in four light irradiation treatments (100, 50, 24 and 12% full sunlight). The photosynthetic capacity, essential oil content and composition, activities of several enzymes and levels of some secondary metabolites involved in the shikimic acid and cinnamic acid pathways were analyzed. The leaf mass per area, average diurnal net photosynthetic rate, and the essential oil content increased significantly with increasing light intensity. Phenylalanine, cinnamic acid, and *p*-coumaric acid in the cinnamic acid pathway were at their highest levels in plants cultivated in 100% full sunlight. The highest content of shikimic acid in the shikimic acid pathway was obtained in plants grown in 50% sunlight transmittance. The activity of the enzymes 3-Deoxy-D-arabino-heptulosonate-7-phosphate synthase, phenylalanine ammonia lyase, cinnamate-4-hydroxylase and 4-coumarate:CoA ligase increased proportionally with light intensity. Overall, we conclude that high light irradiation promotes high net photosynthetic rate, high activity of enzymes and high amounts of phenylpropanoid precursor metabolites leading to significant biosynthesis of essential oil in *A*. *heterotropoides* var. *mandshuricum*.

## Introduction

*Asarum heterotropoides* Fr. Schmidt var. *mandshuricum* (Maxim.) Kitag (Chinese wild ginger) is one of the most exploited native skiophyte medicinal herbs, which is adapted to shade, moist environments in China. This species belonging to the aristolochiaceae family is native to northeast China, Korean Peninsula, Japan, and Russia. It is a low-growing, herbaceous perennial plant producing a cluster of leaves up to 15 cm tall. Fibrous root of *A*. *heterotropoides* var. *mandshuricum* is used in medicine for its anti-inflammatory, anti-bacterial, anti-pyretic, fungistatic and antalgic properties [1–3]. Essential oil extracted from its fibrous roots acts as sedative, incitant and fungicide [4, 5]. As a result, the essential oil is a key indicator for assessing quality of *A*. *heterotropoides* var. *mandshuricum* to be used as medicine.

**Fig 1:**
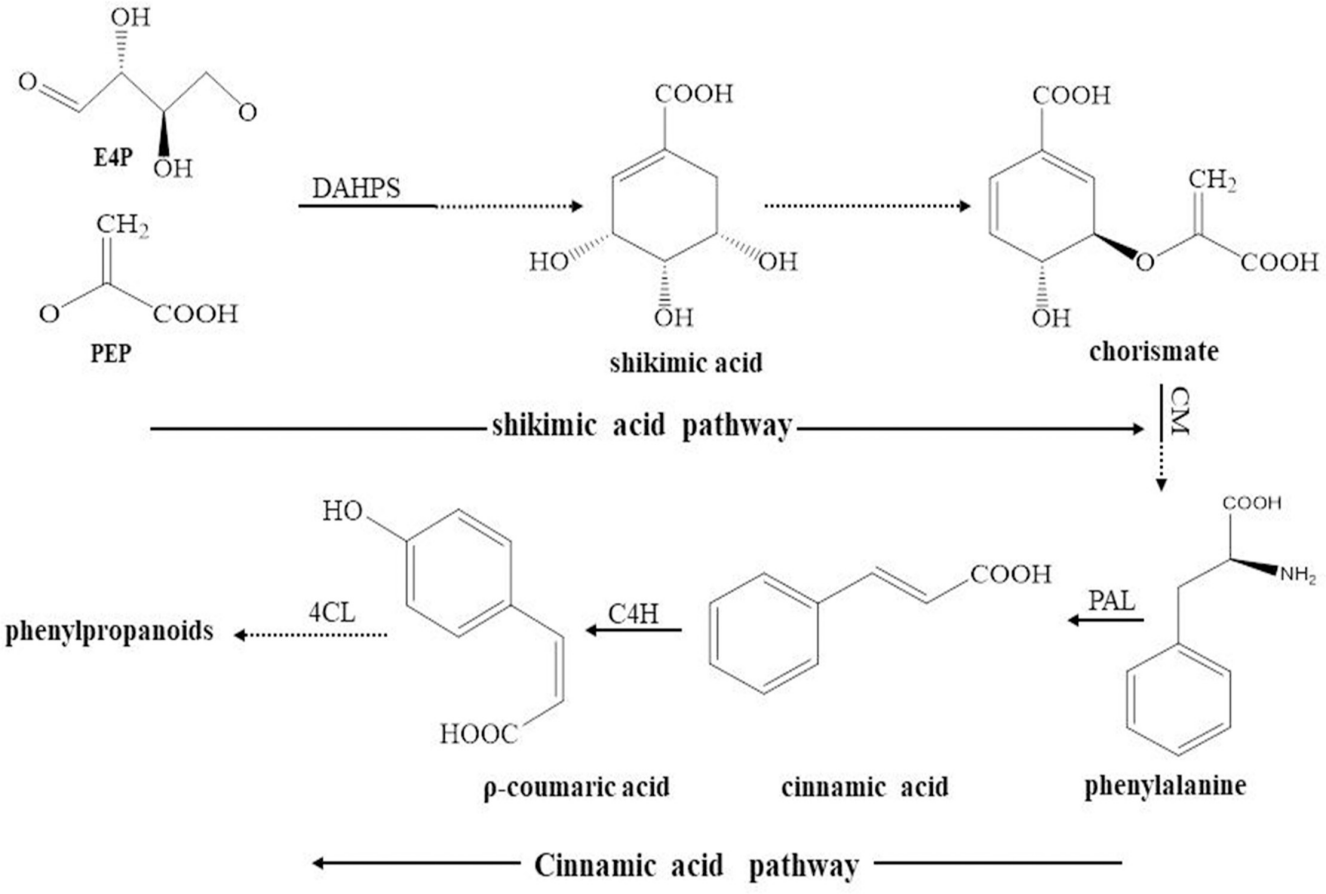
Phenylpropanoid biosynthesis through the shikimic acid and cinnamic acid pathways (referring to Maeda et al. **[7] and Rastogi et al. [10]).** PEP: phosphoenol pyruvate; C4H: cinnamate-4-hydroxylase and 4CL: 4-coumarate:CoA ligase; CM: chorismate mutase; PAL: phenylalanine ammonia lyase; DAHPS: 3-deoxy-D-arabino-heptulosonate-7-phosphate synthase; E4P: erythrose-4-phosphate.

The essential oil derived from *A*. *heterotropoides* var. *mandshuricum* is mainly composed of phenylpropanoid compounds, including benzene, 1,2-dimethoxy-4-(2-propenyl)-, 1,3-Benzodioxole,5-(2-propenyl)- and 1,3-benzodioxole, 4-methoxy-6-(2-propenyl) [5, 6]. Phenylpropanoid biosynthesis is derived from the shikimic acid and cinnamic acid pathways [7] (Fig 1). Shikimic acid provides the seven carbon skeleton and L-phenylalanine is the phenylpropenes’ initial precursor. 3-Deoxy-D-arabino-heptulosonate-7-phosphate synthase (DAHPS) is one of the key enzymes in aromatic amino acid biosynthesis because it catalyzes an aldol condensation of the glycolytic intermediate phosphoenol pyruvate (PEP) and pentose phosphate pathway intermediate erythrose-4-phosphate (E4P) to a seven-carbon six-membered heterocyclic compound (3-dexoy-D-arabinoheptulosonate-7-phosphate (DAHP)) which is the first enzymatic step in the shikimic acid pathway [6–8]. Chorismate mutase (CM) catalyzes the step of phenylalanine biosynthesis and additionally represents a key hinge toward the branch of phenylalanine biosynthesis. Subsequently, phenylalanine is catalyzed by the well-known phenylalanine ammonia lyase (PAL) to cinnamic acid [9]. This is followed by the formation of *p*-coumaric acid from cinnamic acid. These reactions involve the cinnamate-4-hydroxylase (C4H) and 4-coumarate:CoA ligase (4CL). In subsequent reactions, the derivatives phenylpropenes such as eugenol, methyleugenol, safrole are synthesized [10]. DAHPS, CM, PAL, C4H and 4CL are therefore the key enzymes catalyzing the biosynthesis of phenylpropene precursors. Similarly, cinnamic acid and *p*-coumaric acid are crucial secondary metabolites for the biosynthesis of essential oil in *A*. *heterotropoides* var. *mandshuricum*.

Skiophytic plants grow in moist and shaded areas and their photosynthesis is adapted to low illumination [11]. Skyiophytes have shown optimal growth at light intensities between 200–300 μmol photons m^-2^ s^-1^ or 10–15% of full sunlight. At light intensities greater than 500 μmol photons m^-2^ s^-1^, photoinhibitory symptoms have been reported, including a decrease in the rate of photosynthesis [12]. In order to improve the bio-yield and active ingredient content of herbs, economic and effective actions should be carried out during planting according to plant characteristics such as adjusting light intensities [13–16]. The effects of light irradiation levels on essential oil yield and composition in plants such as sage and basil have been well documented. Shading strongly reduced the total essential oil content of basil and inhibited the accumulation of eugenol, linalool and 1,8-cineole [13]. However, moderate shading (45% full sunlight) promoted sage highest essential oil content while full sunlight benefited the biosynthesis of myrcene [17]. Some researchers suggested that shading leads to reduced essential oil content [18], but less attention has been paid to the relation of light irradiation and essential oil composition and precursor metabolites. In addition, how skiophyte plants respond to high light levels with regard to the biosynthesis of essential oil has been less explored. This study was conducted to investigate the effects of light irradiation on photosynthesis and essential oil accumulation and composition in the skiophyte plant *A*. *heterotropoides* var. *mandshuricum*

## Results

### Net photosynthetic rate and leaf mass per area (LMA) of *A*. *heterotropoides var*. *mandshuricum* in different light irradiations

**Fig 2:**
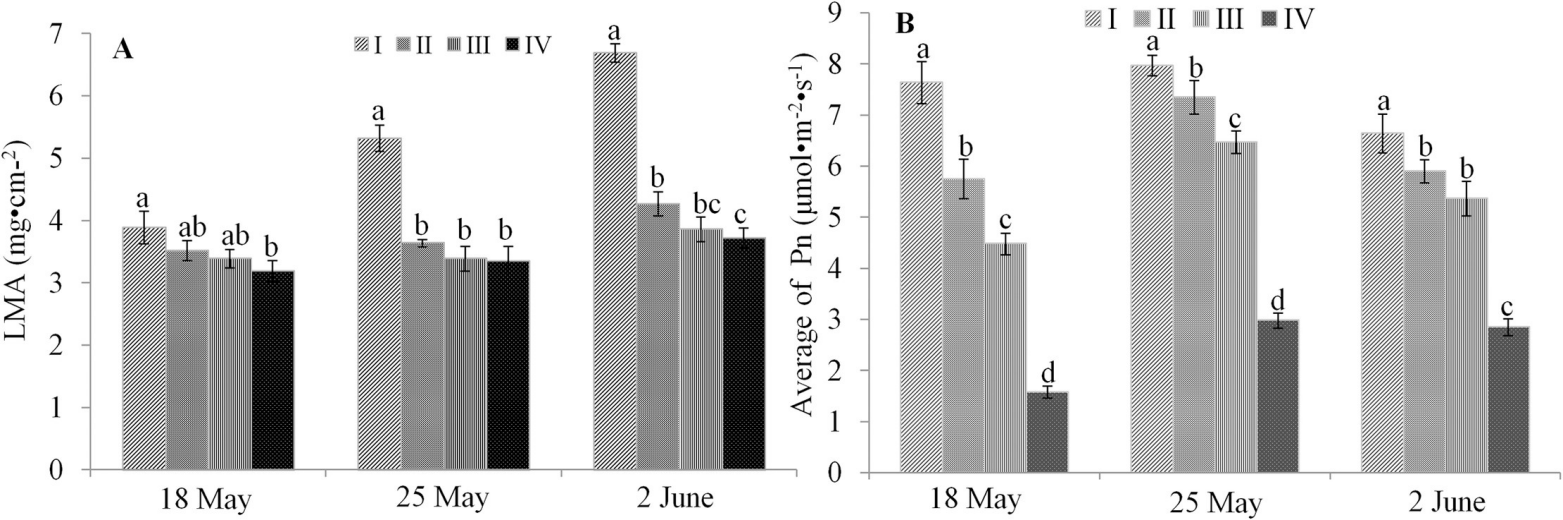
Morphological and physiological responses to light treatments in *A*. *heterotropoides* var. *mandshuricum*. The leaf mass per area (LMA) (A) and average diurnal net photosynthetic rate (Pn) (B) at three phenological stages (18 May, 25 May, 2 June) of *A*. *heterotropoides* var. *mandshuricum* grown in four light radiations. Note: I, 100% full sunlight; II, 50% full sunlight; III, 24% full sunlight; IV, 12% full sunlight. Error bar represents standard deviation (n = 5); different letters on the bars mean significant difference (*P* <0.05).

*A*. *heterotropoides* var. *mandshuricum* were subjected to four treatments: (I) 100%, (II) 50%, (III) 24% and (IV) 12% full sunlight. We measured the diurnal variation of photosynthesis at 18 May, 25 May and 2 June, representing expanding leaf stage, flowering stage and initial fruiting stage, respectively. To verify the effectiveness of our light treatments, we recorded at several time points the photosynthetically active radiation (PAR) which is the amount of light available for photosynthesis. As shown in Table 1, PAR values in the treatments II, III and IV were respectively ~ 50, 24 and 12% of values in the treatment I. LMA is a good indicator for light interception in plant, therefore, we analyzed the LMA of plants from the four treatments [19]. We observed that LMA values of *A*. *heterotropoides* var. *mandshuricum* grown in 100% full sunlight were significantly higher than those in the other three light treatments, particularly at the flowering and initial fruiting stages (Fig 2A). We further investigated the diurnal variation of photosynthesis of *A*. *heterotropoides* var. *mandshuricum* in the four light treatments and calculated the average diurnal net photosynthetic rate (Pn). Our data indicated that the variation trends of Pn in the different light treatments were similar across the three phenological stages (Fig 2B). In addition, Pn values in 100% full sunlight were significantly higher than those in the three other light treatments. Pn values declined gradually with decreasing light irradiation (Fig 2B). Overall, these results suggest that increasing light intensity induces high photosynthetic capacity in leaf of *A*. *heterotropoides* var. *mandshuricum*.

**Table 1:** The available light radiance of different light treatments in *A*. *heterotropoides* var. *mandshuricum*.

Light treatments	Photosynthetically active radiation (μmol•m^-2^•s^-1^)																	
	Time points (h:min) on 18 May						Time points (h:min) on 25 May						Time points (h:min) on 2 June					
	6:30	8:30	10:30	12:30	14:30	16:30	6:30	8:30	10:30	12:30	14:30	16:30	6:30	8:30	10:30	12:30	14:30	16:30
**Ⅰ**	643	1243	1599	1312.5	1103	384	1031	1693	1860	1747	1603	1174	996	1713	1870	1791	1331	1074
**Ⅱ**	323	576	830	713	615	257	530	849	940	893	814	657	488	652	931	870	850	625
**Ⅲ**	173	358	480	392	233	100	269	433	547.5	452	292	172	268	337	514	470	370	217
**Ⅳ**	80	115	165	139	129	40	98	128	262	169	147	97	113	154	260	239	144	115

### Effect of light irradiation on essential oil content and composition in *A*. *heterotropoides var*. *mandshuricum*

**Fig 3:**
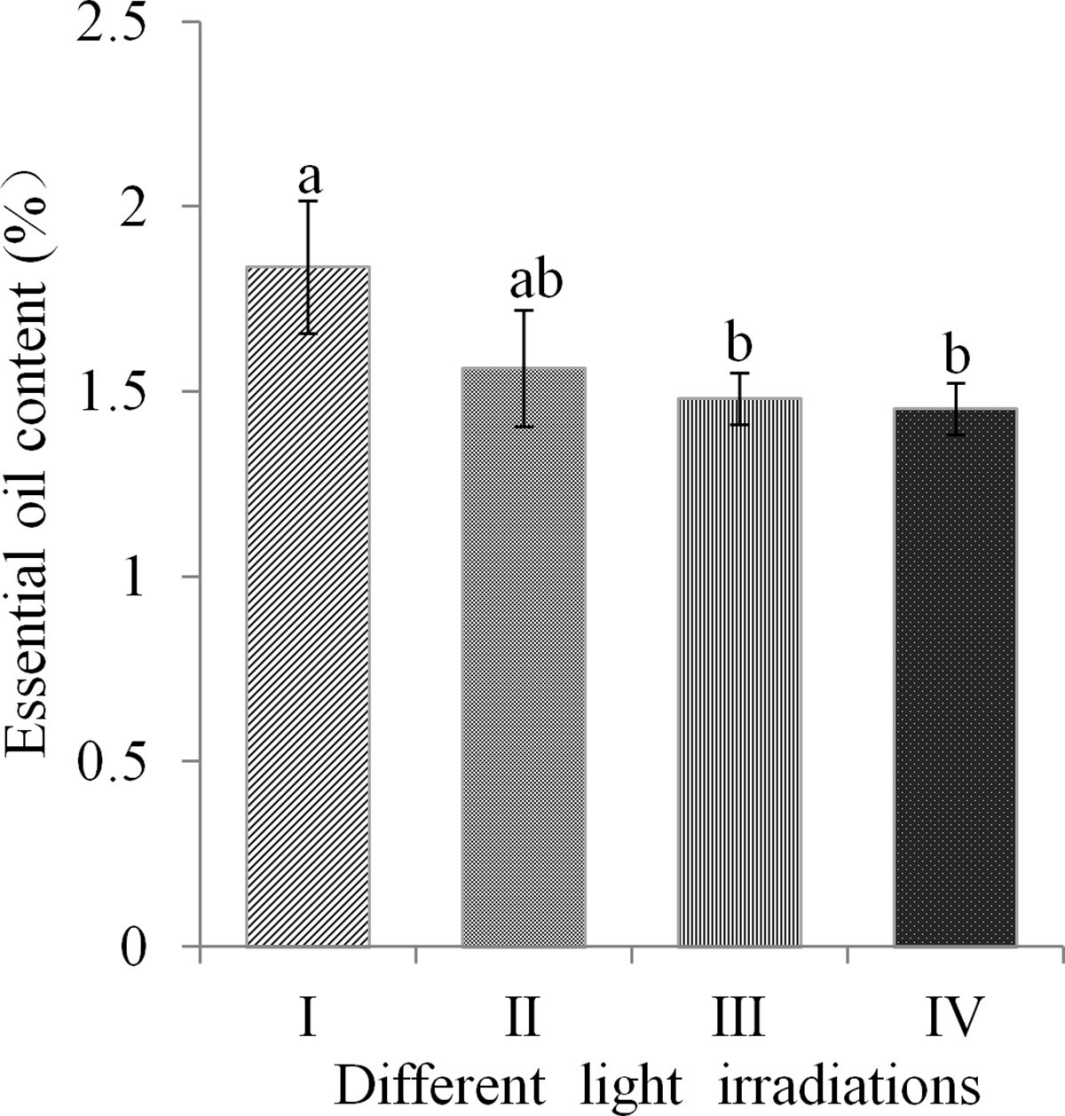
Essential oil content in fibrous roots of *A*. *heterotropoides* var. *mandshuricum* plants grown in different light irradiations. Note: I, 100% full sunlight; II, 50% full sunlight; III, 24% full sunlight; IV, 12% full sunlight. Error bar represents standard deviation (n = 5); different letters on the bars mean significant difference (*P* <0.05).

It has been shown that light treatments affect essential oil yield and composition in plants [13, 19–21]. We extracted the essential oil from fibrous roots of *A*. *heterotropoides* var. *mandshuricum* pants grown in the four light treatments and compared the yield. We observed that the essential oil yield was proportional to the light irradiation (Fig 3). The highest oil yield (1.86%, mL 100 g^-1^) was obtained in fibrous roots of plants grown in 100% full sunlight, while the lowest oil yield (1.30%, mL 100 g^-1^) was obtained from fibrous roots of plants grown in 12% full sunlight (Fig 3). This result shows that high light intensity promotes biosynthesis of essential oil in *A*. *heterotropoides* var. *mandshuricum* roots.

**Fig 4:**
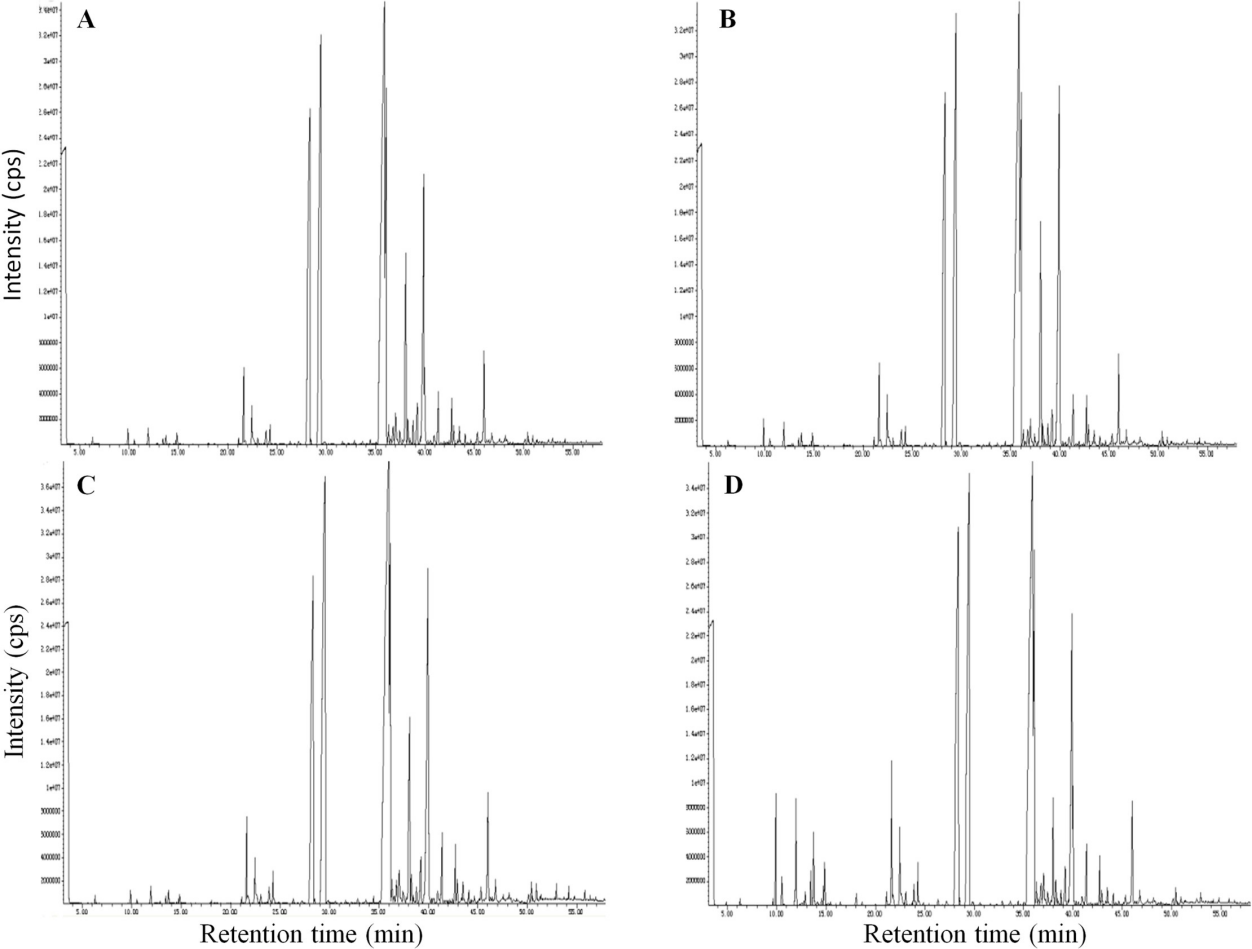
Gas chromatogram of phenylpropanoid and aromatic compounds in essential oil from fibrous roots of *A*. *heterotropoides var*. *mandshuricum* plants grown in four light conditions. A, 100% full sunlight; B, 50% full sunlight; C, 24% full sunlight; D, 12% full sunlight. cps = count per second.

**Fig 5:**
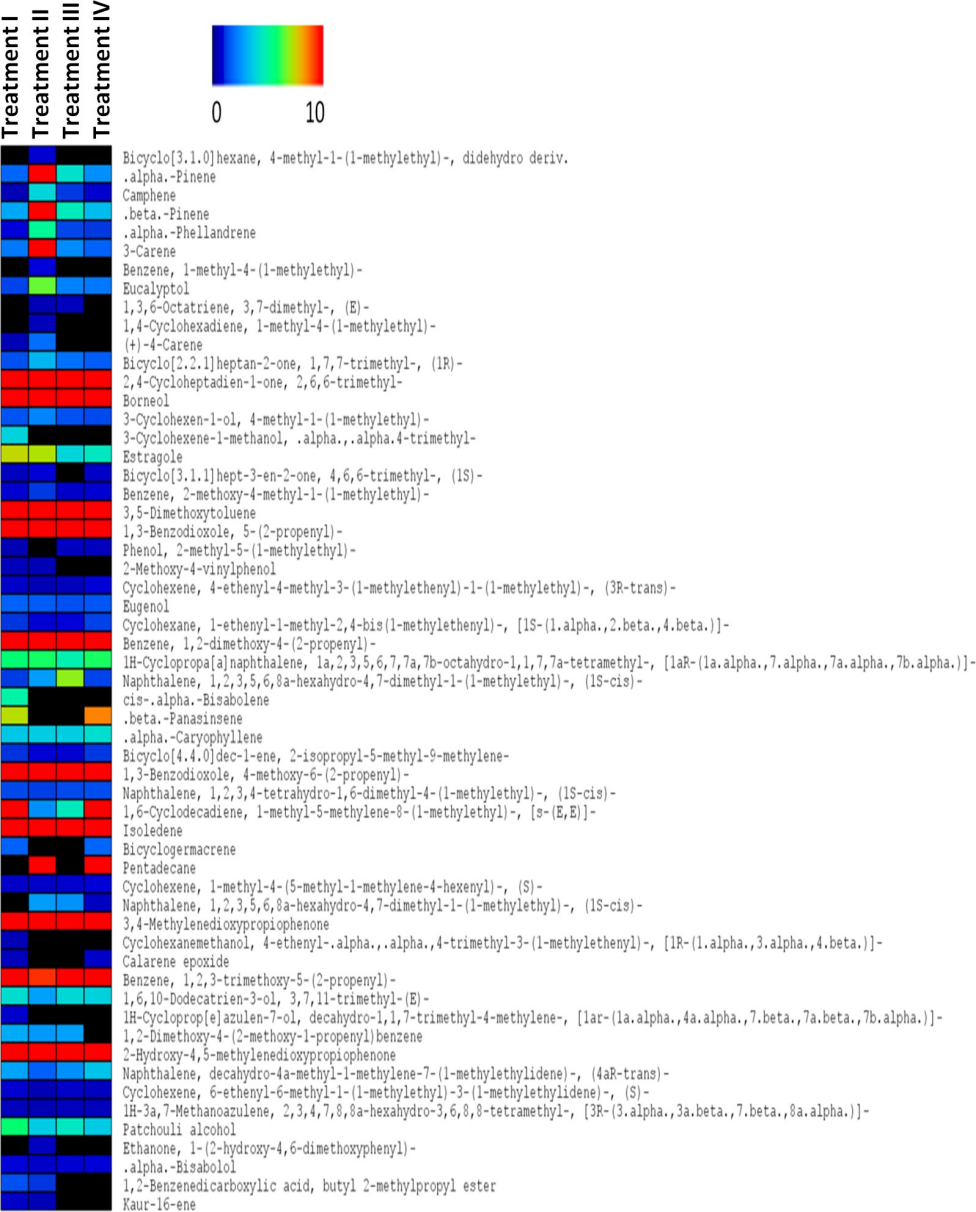
Heatmap displaying the variation of the relative content of phenylpropanoid and aromatic compounds detected in the extracted essential oil from fibrous roots of *A*. *heterotropoides* var. *mandshuricum* grown in four light conditions. Plot colors reflect the proportion of the detected metabolites within the essential oil, ranging from low (black) to high (red). Black means not detected. Treatment I, 100% full sunlight; Treatment II, 50% full sunlight; Treatment III, 24% full sunlight; Treatment IV, 12% full sunlight.

We further analyzed the composition of the esssential oils obtained from the four treatments. According to the GC-MS analysis, essential oil of *A*. *heterotropoides* var. *mandshuricum* was composed of phenylpropanoid compounds, aromatic compounds and terpenoids compounds. The gas chromatogram results of phenylpropanoid and aromatic compounds detected in the four essential oils are displayed in Fig 4. Only compounds with at least 85% similarity to NIST mass spectral library were selected. We identified 50, 50, 41 and 44 compounds in essential oils from plants grown in 100, 50, 24 and 12% full sunlight, respectively (S1 Table). A marked difference was observed in the relative content of these compounds among the four light treatments (Fig 5; S1 Table).

The major phenylpropanoid compounds were 1,3-Benzodioxole,5-(2-propenyl)-, 2-Methoxy-4-vinylphenol, eugenol, Benzene,1,2-dimethoxy-4-(2-propenyl)-, 1,3-Benzodioxole,4-methoxy-6-(2-propenyl)-, 3,4-Methylenedioxypropiophenone, Benzene,1,2,3-trimethoxy-5-(2-propenyl)-, 1,2-Dimethoxy-4-(2-methoxy-1-propenyl)benzene, 2-Hydroxy-4,5-methylenedioxypropiophenone, 1,2-Benzenedicarboxylic acid, and butyl 2-methylpropyl ester. Together, they accounted for 75.5, 66.2, 70.2 and 71.2% of the total essential oil obtained from plants grown in 100, 50, 24 and 12% full sunlight, respectively. Main aromatic compounds detected were Benzene,2-methoxy-4-methyl-1-(1-methylethyl)-, 3,5-Dimethoxytoluene, 2-Methoxy-4-vinylphenol, Phenol,2-methyl-5-(1-methylethyl)-, Ethanone and 1-(2-hydroxy-4,6-dimethoxyphenyl). They occupied 14.8,18.4,15.1 and 14.9% of the total essential oil obtained from plants grown in 100, 50, 24 and 12% full sunlight, respectively. Finally, terpenoids compounds detected in the essential oils include.alpha.-Pinene, Camphene,.beta.-Pinene, 3-Carene,.alpha.-Phellandrene, Eucalyptol, (+)-4-Carene, Bicyclo[2.2.1]heptan-2-one,1,7,7-trimethyl-,(1S)-, 2,4-Cycloheptadien-1-one,2,6,6-trimethyl-, 3-Cyclohexen-1-ol,4-methyl-1-(1-methylethyl)-, Borneol, 3-Cyclohexene-1-methanol, and.alpha.,.alpha.4-trimethyl- which occupied relatively small percentage in the different essential oils (Fig 5; S1 Table). Nonetheless, we observed that the amounts of terpenoid compounds were at their highest levels in 50% full sunlight. Collectively, our data suggest that high light treatments increase essential oil and phenylpropanoid compounds content in *A*. *heterotropoides* var. *mandshuricum* fibous root and the oil composition changes according to the levels of the light intensity.

### Effect of light irradiation on DAHPS, PAL, C4H and 4CL enzyme activity

**Fig 6:**
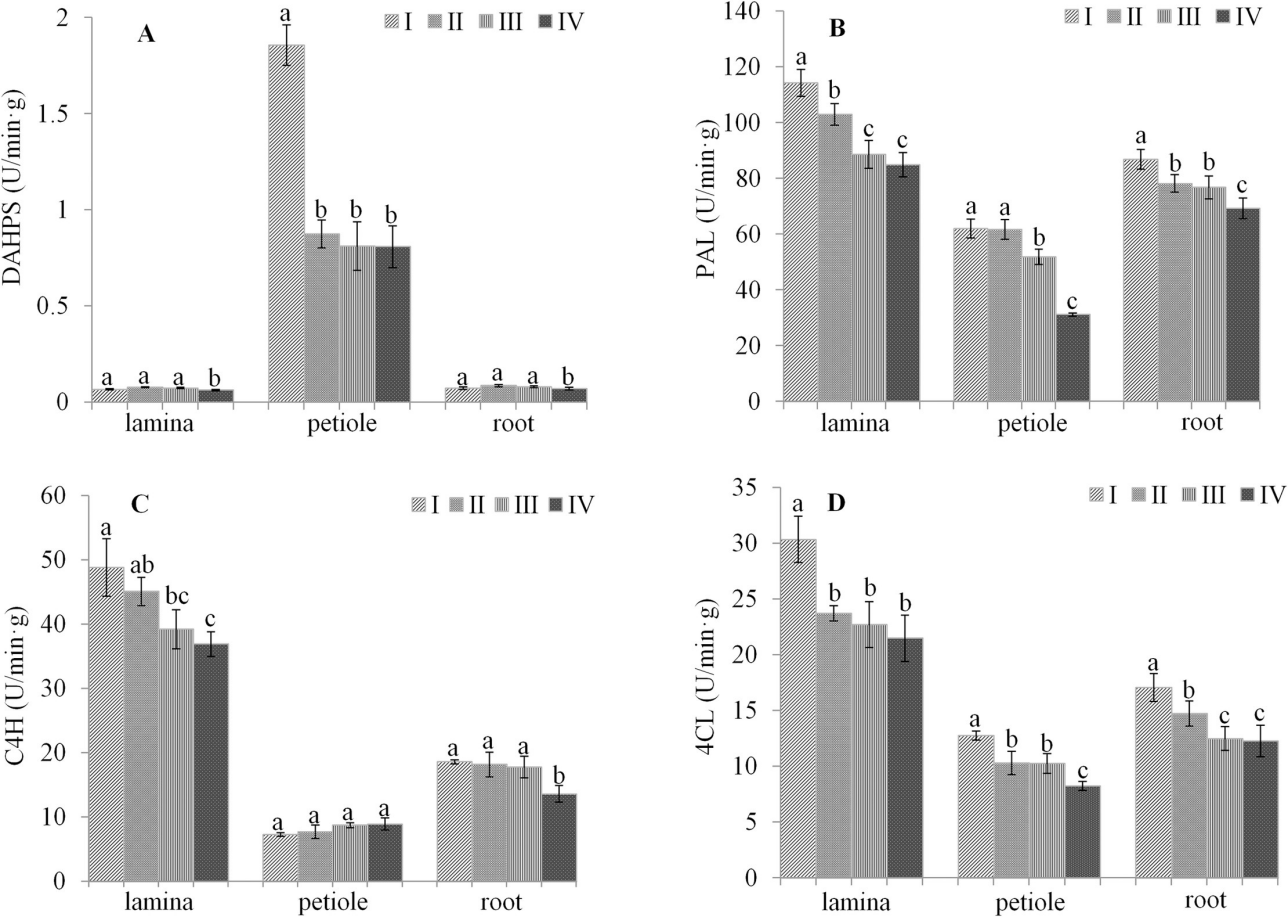
Activity key enzymes involved in the shikimic acid and cinnamic acid pathways. Activity of 3-deoxy-D-arabino-heptulosonate-7-phosphate synthase (DAHPS) (A), phenylalanine ammonia lyase (PAL) (B), cinnamate-4-hydroxylase (C4H) (C), 4-coumarate:CoA ligase (4CL) (D) in different tissues of *A*. *heterotropoides* var. *mandshuricum* grown in four light irradiations. I, 100% full sunlight; II, 50% full sunlight; III, 24% full sunlight; IV, 12% full sunlight. Error bar represents standard deviation (n = 5); different letters on the bars mean significant difference (*P* <0.05).

Key molecules in the essential oil of *A*. *heterotropoides* var. *mandshuricum*. are synthesized through the shikimic acid and cinnamic acid pathways. We examined the activities of four enzymes involved in the shikimic acid and cinnamic acid pathways (Fig 1), including 3-deoxy-D-arabino-heptulosonate-7-phosphate synthase (DAHPS), 4-coumarate:CoA ligase (4CL), cinnamate-4-hydroxylase (C4H) and phenylalanine ammonia lyase (PAL) in the different light treatments in *Asarum* petiole, lamina and fibrous roots (Fig 6). All enzymes were more active in aerial tissues (lamina and petiole) than root. This is understandable since sunlight is mainly percepted by aerial organs (Fig 6). Furthermore, we observed a strong induction of the activity of these enzymes in higher light conditions (100 and 50% full sunlight) in the different tissues (Fig 6). Only C4H activity in petiole was stronger in low light conditions than high light conditions (Fig 6C).

### Secondary metabolites content in plants grown in different light intensities and correlation analysis

**Fig 7:**
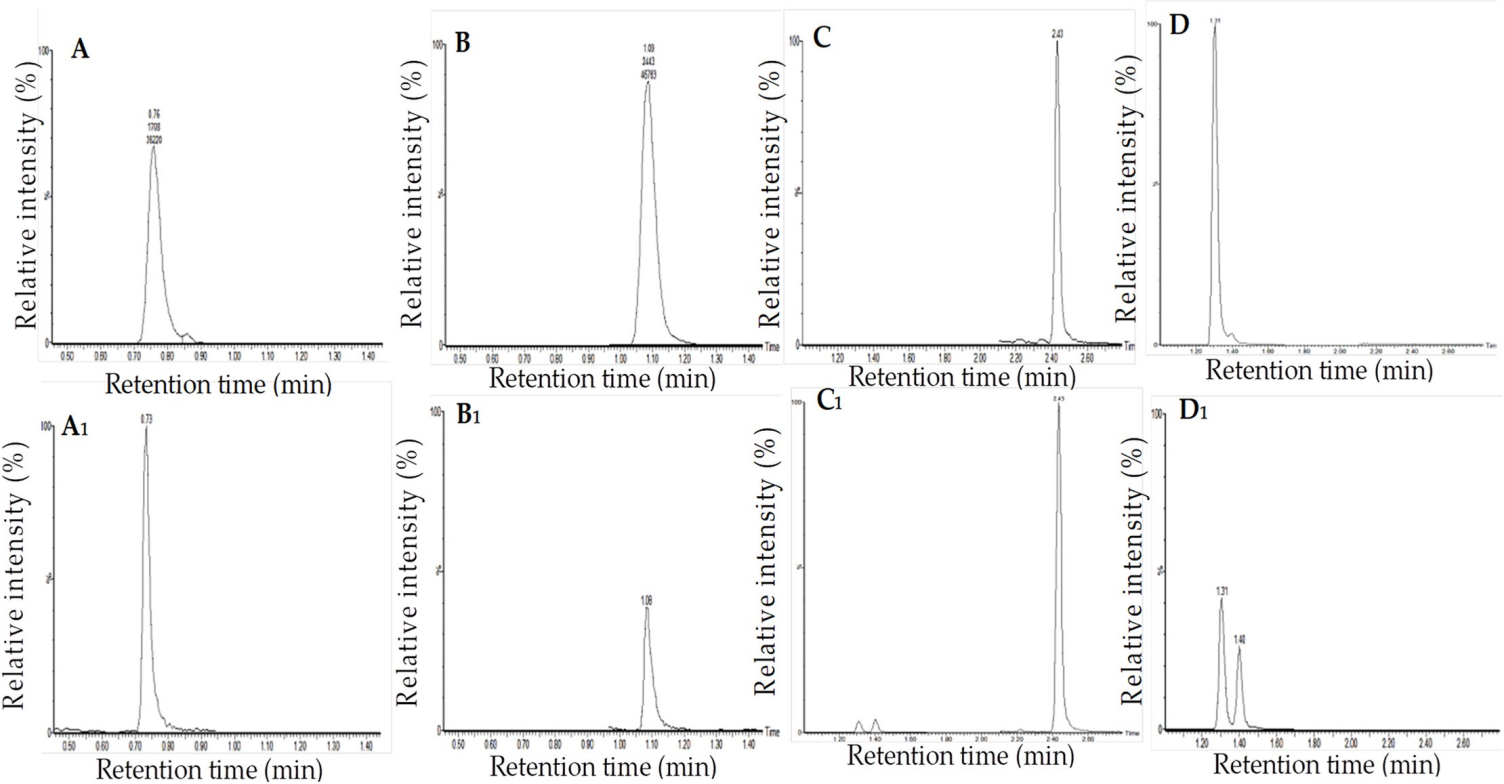
The ion chromatograms of four compounds. (**A**) chromatogram of shikimic acid standard, (**A**_**1**_) chromatogram of shikimic acid in *A*. *heterotropoides var*. *mandshuricum* sample; (**B**) chromatogram of phenylalanine standard, (**B**_**1**_), chromatogram of phenylalanine in *A*. *heterotropoides var*. *mandshuricum* sample; (**C**), chromatogram of cinnamic acid standard, (**C**_**1**_), chromatogram of cinnamic acid in *A*. *heterotropoides var*. *mandshuricum* sample; (**D**), chromatogram of *p-*coumaric acid standard, (**D**_**1**_) chromatogram of *p-*coumaric acid in *A*. *heterotropoides var*. *mandshuricum* sample.

**Fig 8:**
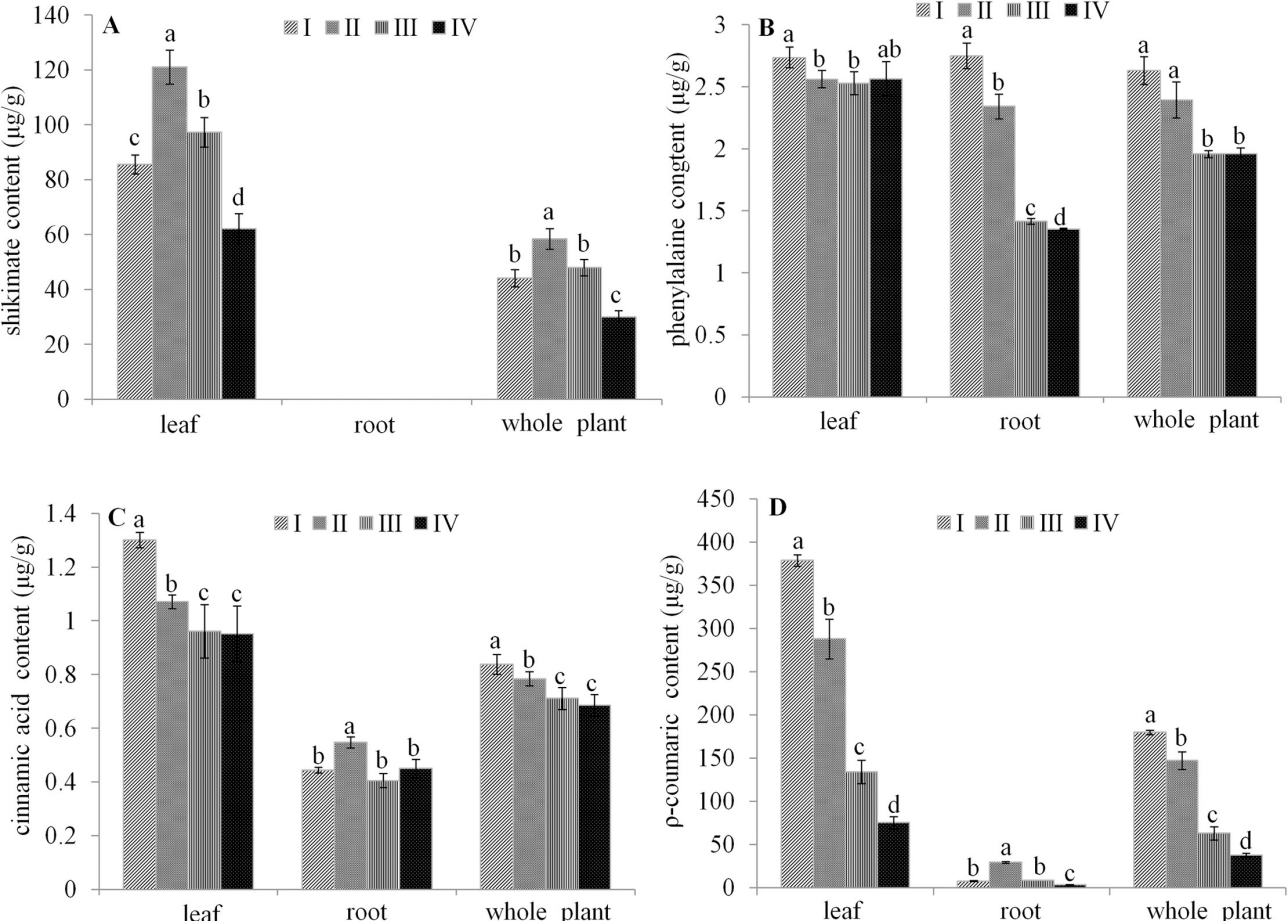
Effect of light treatments of four precursor metabolites involved in the shikimic acid and cinnamic acid pathways. Shikimic acid (A), phenylalanine (B), cinnamic acid (C) and *p*-coumaric acid (D) contents in root, leaf and whole *A*. *heterotropoides* var. *mandshuricum* grown in different light irradiations. Note: I, 100% full sunlight; II, 50% full sunlight; III, 24% full sunlight; IV, 12% full sunlight. Error bar represents standard deviation (n = 5); different letters on the bars mean significant difference (*P* <0.05).

In order to further confirm the positive effect of high light treatments on the shikimic acid and cinnamic acid pathways, we evaluated the accumulation of key precursor metabolites (shikimic acid, phenylalanine, cinnamic acid and *p*-coumaric acid) involved in these pathways in leaf, fibrous root and whole plant samples collected from *A*. *heterotropoides* var. *mandshuricum*. grown in the four light treatments. Fig 7 shows the ion chromatograms of shikimic acid, phenylalanine, cinnamic acid and *p*-coumaric acid. Our results showed that shikimic acid accumulated only in leaf tissues but the other three metabolites were detected in leaves and fibrous roots (Fig 8). In addition, leaf samples had significantly higher contents of the four metabolites than root samples. Overall, we observed that the contents of the four metabolites increased proportionally with the light intensity in both tissues (Fig 8).

We further performed a correlation analysis between light treatments, photosynthetic capacity, essential oil yield and related metabolites in *A*. *heterotropoides* var. *mandshuricum*. Strong and positive correlations were observed between many parameters (Table 2). There were very significant positive correlations between PAR, LMA, Pn, essential oil yield, phenylalanine, cinnamic acid and *ρ*-coumaric acid (*P* < 0.05). However, shikimic acid had no significant correlations with PAR, essential oil, phenylalanine and cinnamic acid. We deduced that high light treatments (high PAR) contibute to high photosynthetic activity (high Pn and LMA), which leads to high accumulation of upstream and downstream phenylpropanoid metabolites (phenylalanine, cinnamic acid and *ρ*-coumaric acid) engaged in the biosynthesis of *A*. *heterotropoides* var. *mandshuricum* essential oil.

**Table 2:** The relationships between PAR, LMA, Pn, essential oil yield and precursor metabolites.

	PAR	LMA	Pn	Essential oil	Shikimic acid	Phenylalanine	Cinnamic acid
LMA	0.977**	1					
Pn	0.853**	0.749**	1				
Essential oil	0.897**	0.917**	0.683*	1			
Shikimic acid	0.314	0.126	0.733**	0.131	1		
Phenylalanine	0.986**	0.961**	0.781**	0.931**	0.222	1	
Cinnamic acid	0.932**	0.864**	0.737**	0.827**	0.218	0.931**	1
ρ-coumaric acid	0.918**	0.805**	0.923**	0.825**	0.601**	0.898**	0.872*

Pn = daily average net photosynthetic rate; PAR = daily average available photosynthetic radiation; LMA = leaf mass per unit area.

** very significant correlation (*P* < 0.01)

* significant correlation (*P* < 0.05).

## Discussion

Leaf mass per area (LMA) is one of the best predictors for light interception in plant [22–25]. In this study, the LMA values increased with increasing light irradiation. This means that leaves of *Asarum heterotropoides* var. *mandshuricum* plants grown in high light conditions can intercept and absorb more light and assimilate more CO_2_ than leaves of plants grown in low light conditions. The net photosynthetic rate (Pn) is known to be an indicator of the light reaction and carbon assimilation [26]. Herein, Pn values increased proportionally with light intensity at three phenological stages showing that the leaves have a strong capacity to assimilate carbon when *A*. *heterotropoides* var. *mandshuricum* is grown in high levels of light irradiation even in 100% full sunlight. This contrasts with other skiophyte plants such as *Panax ginseng* and *Panax quinquefolium* which could not adapt to 100% full sunlight condition [27, 28]. Further physiological investigations are needed to better clarify the mechanisms underlying the adaptation of *A*. *heterotropoides* var. *mandshuricum* to high light conditions. It is well-known that photosynthesis fixes carbon and supplies the original carbon skeleton substance to the biosynthesis of secondary metabolites [29]. The significant positive correlations between PAR, LMA and Pn showed that high light promotes fixation of original carbon skeleton substance in of *A*. *heterotropoides* var. *mandshuricum*.

Essential oil content in many plants is influenced by abiotic factors such as light irradiation [13, 15, 16, 30–33]. Some of these studies focused on the influence of light intensity on the concentration of plant volatile oils [33–35]. Duriyaprapan et al. [33] reported that solar radiation did not influence volatile oil yield and its important components in Japanese mint (*Mentha arvensis* L. subsp. haplocalyx Briquet var. piperascens Holmes). Larissa et al. [34] showed that light treatment did not alter the volatile oil yield of *Ocimum selloi* Benth. Letchamo et al. [35] reported that supplemental light could enhance the volatile oil yield of *Angelica archangelica*. In our study, the highest essential oil content was obtained in roots of plants grown in 100% full sunlight, confirming that high light irradiation promotes the synthesis of essential oil in *A*. *heterotropoides* var. *mandshuricum*. In addition, we showed that the essential oil composition in *A*. *heterotropoides* var. *mandshuricum* was profoundly changed according to the light intensity. Some reports indicated that higher content of terpenoids such as pinene, camphene, 1,8-cineole, trans-caryophyllene is induced by moderate light irradiation [29,31]. In this study, we also found that the amounts of terpenoids were largest in 50% full sunlight grown *A*. *heterotropoides* var. *mandshuricum*. On the other hand, the highest contents of typical phenylpropanoid compounds were obtained in 100% full sunlight in this study. This result indicates that stronger light irradiations promote biosynthesis of phenylpropanoids in *A*. *heterotropoides* var. *mandshuricum*, which is in accordance with reports of Cheng et al. [36]. Since phenylpropanoid compounds account for more than 70% of the total essential oil in *A*. *heterotropoides* var. *mandshuricum*, we infer that the highest essential oil content obtained in roots of *A*. *heterotropoides* var. *mandshuricum* grown in 100% full sunlight is due to a strong biosynthesis of phenylpropanoids. This observation was further supported by the fact that key upstream molecules involved in the phenylpropanoid pathway (phenylalanine, cinnamic acid and *p*-coumaric acid) were all visibly promoted by strong light irradiations. Hence, specific light treatments could be applied to *A*. *heterotropoides* var. *mandshuricum* in order to obtain essential oil enriched with targeted bioactive molecules.

Significant positive correlations between available PAR, LMA, Pn, essential oil content and key precursor metabolites indicate that high carbon assimilation of *A*. *heterotropoides* var. *mandshuricum* in 100% full sunlight stimulates biosynthesis and accumulation of precursor metabolites and essential oil yield. Biosynthesis of secondary metabolites is influenced not only by the amount of original carbon skeleton substance but also by the activities of related enzymes. In this study, we found that strong light irradiations increased the activity levels of DAHPS, PAL, C4H and 4CL. In agreement with our results, numerous studies also showed pronounced induction of the activity levels of these enzymes with increasing light irradiation in various plants [32, 37, 38].

## Conclusions

As an important medicinal plant, increasing key bioactive molecules in *A*. *heterotropoides* var. *mandshuricum* is crucial. Although *A*. *heterotropoides* var. *mandshuricum* is a skiophyte plant species, this research demonstrated that increasing the intensity of light irradiation results in increased leaf mass per area and net photosynthetic rate, indicating that *A*. *heterotropoides* var. *mandshuricum*. is able to adapt to high light intensity. Furthermore, we showed that high light irradiation significantly increased the content of essential oil mainly because of the strong accumulation of phenylpropane compounds. Overall, our findings suggest that high sunlight treatment could be an effective approach for increasing essential oil content and quality in *A*. *heterotropoides* var. *mandshuricum*.

## Materials and methods

### Plant material

*A*. *heterotropoides* var. *mandshuricum* cultivar “zhongnong xixin 1” was used as plant material in this study. When plants became dormant, 1000 plants (4-year-old) were transplanted into 30 x 40 cm (diameter x height) pots containing humus soil (pH = 6.28). Each pot contained five plants and pots were placed at Jilin Agricultural University Medicinal Herbs Garden (43.80’N, 125.42’E). One year later, soon after the leaves appeared, four light treatments were applied. For Treatment I, the plants were exposed to 100% full sunlight in order to receive natural light irradiation during the day. In the other three treatments, the plants were covered with different layers of black nylon shade netting to receive about 50% (II), 24% (III) and 12% (IV) of full sunlight (Table 1). The photosynthesis, leaf mass to leaf area ratio (LMA), essential oil content, precursors content and enzyme activity were measured.

### Photosynthesis and LMA measurement

The diurnal variations of photosynthesis were measured on 18 May, 25 May and 2 June and measurements were taken on five plants every two hours from 6:20 a.m. to 4:50 p.m. using a CIRAS-2 portable photosynthesis system. The leaves from plants in the four treatments were sampled in five replicates at 10:00 a.m. on 18 May, 25 May and 2 June. LMA was calculated using the methods outlined by Mielke et al. [39].

### Extraction and gas chromatography-mass spectrometry (GC-MS) analysis of phenylpropanoid and aromatic compounds in *A*. *heterotropoides var*. *mandshuricum* essential oil

After harvesting on June 2^nd^, fibrous roots of *A*. *heterotropoides* var. *mandshuricum* from the four light treatments were dried in a shaded place, ground to powder and used for essential oil extraction. About 20 g of samples were separately subjected to hydrodistillation for 3 h in accordance with Chinese Pharmacopoeia method and used to determine the yield of essential oil. 10 μL of essential oil was taken from the solution and diluted 50 times using petroleum ether. 1 μL of this diluted sample was analyzed by GC-MS. A HP-5MS highly polar capillary column (30 m × 0.32 mm × 0.25 μm, Hewlett-Packart, Palo Alto, CA, USA) coated with a 100% polyethylene glycol stationary phase was used. The following oven temperatures and times were used: 40°C (held for 2 min), raised from 40°C to 160°C at a rate of 2.5°C min^-1^, from 160°C to 280°C at a rate of 8°C min^-1^ and finally held at 280°C for 10 min. The injection temperature was set at 280°C. Helium was used as the carrier gas with a flow rate of 1.0 mL min^-1^. An injection volume of 1 μL was used with a split ratio of 100:1. The mass spectrometer operated under a mode of electron impact (EI) at 70 eV with the scan ranges between 30 and 550 amu. The ion source temperature was maintained at 230°C and the quadrupole at 150°C.

Compound identification was done by comparing the obtained peaks with NIST library reference of mass spectral library. The compounds with > 85% similarity to NIST mass spectral library were kept. Relative percentage composition of the compounds was computed from gas chromatography peak areas with DB-5ms column without applying correction factors [40].

### Enzyme assays

#### DAHPS activity

DAHPS activity was assayed as described by Li et al. [41] with some modifications. Fresh leaves frozen with liquid nitrogen were homogenized with a mortar and a pestle on ice in 3 mL pre-cooled 50 mM Tris-HCL buffer (pH = 7.4) containing 1.4 mM 2-mercaptoethanol, 0.1 mM phenylmethysulfonyl fluoride, 1% polyvinylpyrrolidone (M/V), and 10 μM leupeptin. The homogenate was kept at 4°C for 30 min and then centrifuged at 12000 × g for 20 min. The supernatant was used for the assay. The assay mixture contained 0.8 mL extract and 2.2 mL of 50 mM Tris-HCl buffer (pH = 7.5) containing 0.2 mM phosphoenolpyruvate, 0.1 mM erythrose-4- phosphate, and 0.1 mM MnSO_4_/0.1 mM CoCl_2_. The mixtures were incubated at 30°C for 30 min. The reaction was initiated by the addition of enzyme and terminated by the addition of 500 μL of 25% (W/V) trichloroacetic acid. Reaction mixtures without enzyme were run in parallel and used as controls. One unit of activity was defined as the amount of enzyme that catalyzed the synthesis of 1 nmol of DAHPS per minute at 30°C. The concentration of DAHPS was calculated at 549 nm using a molar extinction coefficient of 45000 M^−1^ cm^−1^.

#### PAL activity

PAL activity was assayed as described by Vannelli et al. [38] with some modifications. Fresh leaves frozen with liquid nitrogen were homogenized on ice in 3 mL pre-cooled 0.1 M sodium borate buffer (pH = 8.8) containing 5 mM 2-mercaptoethanol, 1 mM EDTA, and 0.4% polyvinylpyrrolidone (M/V). The homogenate was kept at 4°C for 30 min and then centrifuged at 12000 × g for 20 min. The supernatant was used for the assay. The assay mixture contained 0.8 mL extract and 2.2 mL of 0.1 M sodium borate buffer (pH = 8.8) containing 120 μM L-Phe. The mixtures were incubated at 25°C for 40 min. The reaction was terminated by the addition of 120 μL of 6 N HCL. Reaction mixtures without enzyme were run in parallel and used as controls. The reaction was then followed by monitoring the absorbance of the product, trans-cinnamic acid, at 290 nm using a molar extinction coefficient of 9000 M^−1^ cm^−1^. One unit of activity indicates deamination of 1.0 nmol of L-phenylalanine to CA per minute.

#### C4H activity

C4H activity was assayed as described by Gao et al. [42] with some modifications. Fresh leaves frozen with liquid nitrogen were homogenized on ice in 3 mL pre-cooled 0.05 M Tris-HCL buffer (pH = 8.9), 1 mM phenylmethylsulfonyl fluoride, 15 mM 2-mercaptoethanol, 10 μM leupeptin, 5 mM vitamin C, 0.15% polyvinylpyrrolidone (m/v) 4 mM MgCl_2_, 10% glycerinum. The homogenate was kept at 4°C for 30 min and then centrifuged at 12000 × g for 20 min. The supernatant was used for the assay. The assay mixture contained 0.8 mL extract and 2.2 mL of 0.05 M Tris-HCL buffer (pH = 8.9) containing 2 mM trans-cinnamate, 2 μM NADPNa_2_, and 5 μM Glc-6-phosphate. The mixtures were incubated at 25°C for 30 min. The reaction was terminated by the addition of 100 μL of 6 N HCL. Reaction mixtures without enzyme were run in parallel and used as controls. The reaction was then followed by monitoring the absorbance of the product, 4-hydroxy-trans-cinnamic acid at 340 nm using a molar extinction coefficient of 22600 M^−1^ cm^−1^. One unit of activity indicates deamination of 1.0 nmol product per min.

#### 4CL activity

4CL activity was assayed as described by Knobloch et al. [43] and Lee et al. [44] with some modifications. Fresh leaves frozen with liquid nitrogen were homogenized on ice in 3 mL pre-cooled 0.2 M Tris-HCl (pH = 7.8), 15 mM 2-mercaptoethanol, 5 mM EDTA, 10 μM leupeptin, 1 mM phenylmethylsulfonyl fluoride, 0.15% polyvinylpyrrolidone (m/v), and 30% glycerinum. The homogenate was kept at 4°C for 30 min and then centrifuged at 12000 × g for 20 min. The supernatant was used for the assay. The assay mixture contained 0.8 mL extract and 2.2 mL of 0.2 M Tris-HCL buffer (pH = 7.8) containing 5 mM MgSO_4_, 5 mM ATP, 0.2 mM CoASH, and 0.2 mM *p*-coumarate. The mixtures were incubated at 30°C for 30 min. The reaction was terminated by the addition of 20 μL of 20% formic acid. Reaction mixtures without enzyme were run in parallel and used as controls. The reaction was then followed by monitoring the absorbance of the product, 4-coumaroyl-CoA, at 333 nm using a molar extinction coefficient of 21000 M^−1^ cm^−1^. One unit of activity indicates deamination of 1.0 nmol product per minute.

### Analysis of four precursor metabolites

#### Standards

Standards of shikimic acid (EC: 205-334-2), phenylalanine (EC: 200-568-1), cinnamic acid (EC: 205-398-1) and *p*-coumaric acid (CAS: 588-30-7) were purchased from Sigma-Aldrich (Shanghai) Trading Co. Ltd.

#### Preparation of extracts

After harvesting, plant materials were oven-dried at 40°C and ground to fine powder for measuring contents of shikimic acid, phenylalanine, cinnamic acid and *p*-coumaric acid. Extracts of materials were prepared according to the methods described by Elzaawely et al. [45] with some modifications. About 5 g of samples were boiled in 50 mL distilled water for 55 min. After cooling at room temperature, the water extracts from samples were filtered and extracted with hexane (3×50 mL). One part of the aqueous solutions after extraction with hexane was hydrolyzed with 25 mL 4 M NaOH at 50°C by stirring for 4 h. The suspensions were separately filtered and pH was adjusted to 2.0 by 4 M HCL. Afterwards, the filtrates were separately extracted with ethylacetate (3×50 mL) and then filtered. The ethylacetate extracts were separately dried under vacuum at 40°C. The other part of the aqueous solutions after extraction with hexane remained as the aqueous phase. The aqueous phase and ethylacetate phase dissolved with methanol (Fisher, chromatography grade) were subjected to HPLC-MS/MS analysis.

#### UPLC-MS/MS analysis of the four secondary metabolites

Chromatographic grade acetonitrile and methanol were purchased from Fisher (USA). Standard solutions of all metabolites were prepared by dissolving precisely weighed samples in a precise volume of methanol. The detection of the metabolites was performed using an ultra-performance liquid chromatography system (Waters Acquity UPLC) coupled with a quadrupole mass spectrometers (Waters Xevo TQ) equipped with an ESI source. Calibration curves, method validation, linearity, accuracy and precision, extraction recovery, stability etc., were performed as previously described by Zhang et al. [46] with some modifications. The chromatographic separations of shikimic acid and phenylalanine were carried out over an HSS T3 Column 2.1×100 (mm, id, particle size 1.8 μm) using an isocratic elution mode. The mobile phase consisted of 25% acetonitrile (containing 0.25‰ formic acid) and 75% water (containing 0.25‰ formic acid). The detection was performed in negative modes. The capillary voltage was 2.8 kV with desolvation temperature at 450°C. The chromatographic separations of cinnamic acid and *p*-coumaric acid were carried out over an HSS T3 Column 2.1×100 (mm, id, particle size 1.8 μm) and eluted using an isocratic mobile phase consisted of 15% acetonitrile (containing 0.25‰ formic acid) and 85% water (containing 0.25‰ formic acid). The detection was performed in positive ion mode and the capillary and voltage (ESI) was 3.0 kV with desolvation temperature 450°C. The precursor-product ion pairs, fragmentor voltage (Frag) and collision energy (CE) for the analytes were as follows: 93.01/ 93.0193.0 m/z Frag 22 V; CE 12 V, 8 V for shikimic acid, 93.01/93.0193.01.01 m/z Frag 26 V; CE 14 V, 12 V for phenylalanine, 103.04/131.04 m/z Frag 18 V; CE 20 V, 10 V for cinnamic acid, m/z 93.00/3119.33 Frag 22 V; CE 24 V, 28 V for *p*-coumaric acid. Dwell times of shikimic acid, phenylalanine, cinnamic acid and *p*-coumaric acid were all 16 ms. The flow rate of analyses was 0.5 mL min^-1^. The column temperature was maintained at 35°C and the injection volume was 2 μL. The data were analyzed using the Masslynx software.

### Statistical analysis

All experiments were repeated five times. Data were analyzed using a one-way Analysis of Variance in SPSS statistical software version 22.0. Tests of significant differences among treatments were analyzed using the Least Significant Difference (LSD) test. The significance level was set at *P* < 0.05. The correlation analyses were carried out using the Pearson’s correlation coefficients test.

## Supporting information

S1 Table Phenylpropanoid and aromatic compounds of *Asarum heterotropoides var*. *mandshuricum* essential oil obtained from fibrous roots of plants grown in four light conditions. I, 100% full sunlight; II, 50% full sunlight; III, 24% full sunlight; IV, 12% full sunlight.
(XLSX)

## Abbreviations

DAHPS: 3-deoxy-D-arabino-heptulosonate-7-phosphate synthase
C4H: Cinnamate-4-hydroxylase
4CL: 4-coumarate:CoA ligase
CM: Chorismate mutase
CA: Cinnamic acid
E4P: Erythrose-4-phosphate
LMA: Leaf mass per area
PEP: Phosphoenol pyruvate
Pn: net photosynthetic rate
PAL: Phenylalanine ammonia lyase
PAR: Photosynthetically active radiation

## References

[1] Drew A.K.; Whyte I.M.; Bensoussan A.; Dawson A.H.; Zhu X.; Myers S.P. Chinese herbal medicine toxicology database: monograph on Herba Asari, “xi xin”. *J*. *Toxicol*. *Clin*. *Toxicol*. 2002, 40, 169–172. 10.1081/clt-120004405 12126188

[2] Zhu Y.P. Chinese Materia Medica; Chemistry Pharmacology and Applications. 1988, Florida, USA: CRC Press.

[3] Huang J.; Wang H.Q.; Zhang C.; Li G.Y.; Lin R.C.; Wang J.H. A new tetrahydrofuran-type lignan with anti-inflammatory activity from *Asarum heterotropoides* Fr. Schmidt var *mandshuricum*. *J*. *Asian Nat*. *Prod*. *Res*. 2013, 16, 387–392. 10.1080/10286020.2013.820713 23909403

[4] Hashimoto K; Yanagisawa T.; Okui Y.; Ikeya Y.; Maruno M.; Fujita T. Studies on anti-allergic components in the roots of *AsiAsarum sieboldi*. *Planta Med*. 1994, 60, 124–127. 10.1055/s-2006-959432 8202562

[5] Haque A.S.M.T.; Moon J.N.; Saravana P.S.; Tilahun A.; Chun B.S. Composition of *Asarum heterotropoides* var. *mandshuricum* radix oil from different extraction methods and activities against human body odor-producing bacteria. *J*. *Food Drug Anal*. 2016, 24, 813–821. 10.1016/j.jfda.2016.04.006 28911620 PMC9337282

[6] Sangwan N.S.; Farooqi A.H.A.; Sangwan R.S.; Shabih F. Regulation of essential oil production in plants. *Plant Growth Regul*. 2001, 34, 3–21.

[7] Maeda H.; Dudareva N. The shikimic acid pathway and aromatic amino acid biosynthesis in plants. *Annu*. *Rev*. *Plant Biol*. 2012, 63, 73–105. 10.1146/annurev-arplant-042811-105439 22554242

[8] Tzin V.; Galili G. New insights into the shikimic acid and aromatic amino acids biosynthesis pathway in plants. *Mol*. *Plant* 2010, 3, 956–972. 10.1093/mp/ssq048 20817774

[9] Sadeghi M.; Dehghan S.; Fischer R.; Wenzel U.; Vilcinskas A.; Kavousi H.R.; et al Isolation and characterization of isochorismate synthase and cinnamate 4-hydroxylase during salinity stress, wounding, and salicylic acid treatment in *Carthamus tinctorius*. *Plant Signal*. *Behav*. 2013, 8, e27335 10.4161/psb.27335 24309561 PMC4091385

[10] Rastogi S.; Kumar R.; Chanotiya C.S. 4-Coumarate:CoA ligase partitions metabolites for eugenol biosynthesis. *Plant Cell Physiol*. 2013, 54, 1238–1252. 10.1093/pcp/pct073 23677922

[11] Mache R. and Loiseaux S. Light saturation of growth and photosynthesis of the shade plant *Marchanitia polymorpha*. *J*. *Cell sci*.1973, 12, 391–401. 4704634 10.1242/jcs.12.2.391

[12] Miskell J.A., Parmenter G. and Eaton-Rye J. J. Decreased Hill reaction rates and slow turnover of transitory starch in the obligate shade plant *Panax quinquefolius* L. (American ginseng). *Planta* 2002, 215,969–979 10.1007/s00425-002-0839-9 12355157

[13] Lee B.S.; Seo B.S.; Chung S.J. Shading effects on growth and essential oil content of hydroponically grown sweet basil (*Ocimum basilicum* L.). *J*. *Kor*. *Soc*. *Hortic*. *Sci*. 1994, 35, 95–102.

[14] Morelli G.; Ruberti I. Light and shade in the photocontrol of Arabidopsis growth. *Trends Plant Sci*. 2002, 7, 399–404. 10.1016/s1360-1385(02)02314-2 12234731

[15] Chang X.M.; Alderson P.G.; Wright C.J. Solar irradiance level alters the growth of basil (*Ocimum basilicum* L.) and its content of volatile oils. *Environ*. *Exp*. *Bot*. 2008, 63, 216–223.

[16] Peralta G.; Pérez-Looréns J.L.; Hernández I.; Vergara J.J. Effects of light availability on growth, architecture and nutrient content of the seagrass *Zostera noltii* Hornem. *J*. *Exp*. *Mar*. *Biol*. *Ecol*. 2002, 269, 9–26.

[17] Li, Y.; Craker, L.E.; Potter, T. Effect of light level on essential oil production of sage (*Salvia officinalis*) and thyme (*Thymus vulgaris*). In: Proceedings of the International Symposium on Medicinal and Aromatic Plants. *Acta Hortic* 1996, *426*, 419–426.

[18] Fernandes V.F.; Almeida B.; Emily V.R.; Feijó S.; Silva D.C.; Oliveira R.A.; Mielke M.S.; Costa L.C. Light radiation on growth, leaf micromorphology and essential oil production of Ocimum gratissimum. *Revista Brasileira de Farmacognosia*. 2013, 23, 419–424.

[19] Wang Y.; Guo S.; Cao J.; Pang X.; Zhang Z.; Chen Z.; Zhou Y.; Geng Z.; Sang Y.; Du S. Toxic and Repellent Effects of Volatile Phenylpropenes from *Asarum heterotropoides* on *Lasioderma serricorne* and *Liposcelis bostrychophila*. *Molecules* 2018, 23(9), 2131. 10.3390/molecules23092131 PMC6225349 30149520

[20] Wang D.; Wang X.; Xia X. Analysis of season variation of methyleugenol and safrole in *Asarum heterotropoides* by gas chromatography. *Chinese Journal of Chromatography* 1997, 15, 85–86. 15739449

[21] Dan Y; Liu HY; Gao WW; Chen SL. Activities of essential oils from *Asarum heterotropoides* var. *mandshuricum* against five phytopathogens. *Crop Protection* 2010, 29, 295–299.

[22] Chazdon R.L.; Kaufmann S. Plasticity of leaf anatomy of two rain forest shrubs in relation to photosynthetic light acclimation. *Funct*. *Ecol*. 1993, 7, 385–394.

[23] Niinemets Ü.; Tenhunen J.D. A model separating leaf structural and physiological effects on carbon gain along light gradients for the shade-tolerant species *Acer saccharum*. *Plant Cell Environ*. 1997, 20, 845–866.

[24] Rosati A.; Badeck F.W.; De Jong T.M. Estimating canopy light interception and absorption using leaf mass per unit leaf area in *Solanum melongena*. *Ann*. *Bot*. 2001, 88, 101–109.

[25] Fila G.; Sartorato I. Using leaf mass per area as predictor of light interception and absorption in crop/weed monoculture or mixed stands. *Agr*. *Forest Meterorol*. 2011, 151, 575–584.

[26] Baker N.R. Chlorophyll fluorescence: A probe of photosynthesis in vivo. *Annu*. *Rev*. *Plant Biol*. 2008 59, 89–113. 10.1146/annurev.arplant.59.032607.092759 18444897

[27] Soc J.M.; Fournier A.R.; Gosselin A.; Procter J.T.A.; Gauthier L.; Khanizadeh S.; Dorais M. Relationship between understory light and growth of forest-grown American-ginseng (*Panax quinquefolius* L.). *J*. *Am*. *Soc*. *Hortic Sci*. 2004, 129, 425–432.

[28] Xu K.Z.; Cao Z.; Zhang W.; Xiu L. Studies on the photosynthetic characteristics of Chinese *Panax ginseng* leaves. *Sci*. *Agric*. *Sin*. 1990, 23, 69–74.

[29] Lewis C.E., Noctor G., Causton D., Foyer C. H. Regulation of assimilate partitioning in leaves. *Aust*. *J*. *Plant Physiol*. 2000, 27, 507–519.

[30] Feijó E.V.R.; Oliveira R.A.; Costa L.C. Light affects *Varronia curassavica* essential oil yield by increasing trichomes frequency. *Rev*. *Bras*. *Farmacogn*. 2014, 24, 516–523.

[31] Pacheco V.F.; Avelara P.R.; Alvarenga I.C.A.; Bertolucci S.K.V.; Alvarenga A.A.; Pintoa J.E.B.P. Essential oil of monkey-pepper (*Piper aduncum* L.) cultivated under different light environments. Ind. *Crops and Prod*. 2016, 85, 251–257.

[32] Xu Y.; Wang G.B. Light radiation affects the growth and flavonol biosynthesis of Ginkgo (*Ginkgo biloba* L). *New Forests* 2014, 45, 765–776

[33] Duriyaprapan S., Britten E. J. The effect of solar radiation on plant growth oil and oil quality of Japanese Mint. *J*. *Exp*. *Bot*. 1982, 33, 1319–1324.

[34] Letchamo W.; Gosselin A.; Heolzl J. Growth and essential oil content of *Angelica archiangelica* as influenced by light intensity and growing media. *J*. *Essential Oil Res*. 1995, 7, 497–504

[35] Larissa C.; Costa B.; José E.B.; Pinto P.; Castro E.M.; Alves E.; Rosal L.F.; Bertolucci S.K.V.; Alves P.B.; Evangelino T.S. Yield and composition of the essential oil of *Ocimum selloi* Benth. cultivated under colored netting. *J*. *Essential Oil Res*. 2010, 22, 34–39.

[36] Cheng S.H.; Fu X.M.; Mei X.; Zhou Y.; Du B. Watanabe N.; Yang Z.Y. Regulation of biosynthesis and emission of volatile phenylpropanoids/benzenoids in petunia× hybrida flowers by multi-factors of circadian clock, light, and temperature. *Plant Physiol*. *Biochem*. 2016, 107, 1–8. 10.1016/j.plaphy.2016.05.026 27235646

[37] Mori T.; Sakurai M.; Sakuta M. Changes in PAL, CHS, DAHP synthase (DS-Co and Ds-Mn) activity during anthocyanin synthesis in suspension culture of *Fragaria ananassa*. *Plant Cell Tiss*. *Organ Cult*. 2000, 62, 135–139.

[38] Vannelli T.; Xue Z.X.; Breinig S.; Qi W.W.; Sariaslani S.F. Functional expression in *Escherichia coli* of the tyrosine-inducible tyrosine ammonia-lyase enzyme from yeast *Trichosporon cutaneum* for production of *p*-hydroxycinnamic acid. *Enzyme Microb*. *Technol*. 2007, 41, 413–422.

[39] Mielkei MS.; Schaffer B. Leaf gas exchange, chlorophyll fluorescence and pigment indexes of *Eugenia uniflora* L. in response to changes in light radiation and soil flooding. *Tree Physiol*. 2010, 30, 45–55. 10.1093/treephys/tpp095 19923194

[40] Bhuiyan M.N.; Begum J.; Bhuiyan M. Analysis of essential oil of eaglewood tree (*Aquilaria agallocha* Roxb.) by gas chromatography mass spectrometry. *Bangladesh J*. *Pharmacol*. 2009, 4, 24–28.

[41] Li P.P.; Li D.F.; Liu D.; Liu Y.M.; Liu C.; Liu J.S. Interaction between DAHP synthase and chorismate mutase endows new regulation on DAHP synthase activity in *Corynebacterium glutamicum*. *Appl*. *Microbiol*. *Biotechnol*. 2013, 97, 10373–10380 10.1007/s00253-013-4806-0 23467831

[42] Gao H.; Zhang Z.K.; Lv X.G.; Cheng N.; Peng B.Z.; Cao W. Effect of 24-epibrassinolide on chilling injury of peach fruit in relation to phenolic and proline metabolisms. *Postharvest Biol*. *Technol*. 2016 111, 390–397.

[43] Knobloch K.H.; Hahlbrock K. Isoenzymes of p-Coumarate:CoA Ligase from cell suspension cultures of *Glycine max*. *Eur*. *J*. *Biochem*. 1975, 52, 311–320. 10.1111/j.1432-1033.1975.tb03999.x 240682

[44] Lee D.; Meyer K.; Chapple C.; Douglasa C.J. Antisense suppression of 4-coumarate:coenzyme A ligase activity in Arabidopsis leads to altered lignin subunit composition. *Plant Cell* 1997, 9, 1985–1998. 10.1105/tpc.9.11.1985 9401123 PMC157052

[45] Elzaawely A.A.; Xuan T.D.; Tawata S. Essential oils, kava *pyrones* and phenolic compounds from leaves and rhizomes of *Alpinia zerumbet* (Pers.) B.L. Burtt. & R.M. Sm. and their antioxidant activity. *Food Chem*. 2007, 103, 486–494.

[46] Zhang X.X.; Gao L.; Zhang Z.J.; Tian Y. Separation and determination of acetyl-glutamine enantiomers by HPLC–MS and its application in pharmacokinetic study. *J*. *Pharm*. *Analysis* 2017, 7, 303–308. 10.1016/j.jpha.2017.06.003 PMC5790696 29404053

